# Intraoperative Effect of 2D vs 3D Fluoroscopy on Quality of Reduction and Patient-Related Outcome in Calcaneal Fracture Surgery

**DOI:** 10.1177/1071100720926111

**Published:** 2020-06-09

**Authors:** Jens A. Halm, M. Suzan H. Beerekamp, Robert Jan de Muinck-Keijzer, Ludo F. M. Beenen, Mario Maas, J. Carel Goslings, Tim Schepers

**Affiliations:** 1 Trauma Unit, Amsterdam University Medical Centers, Location AMC, Amsterdam, The Netherlands; 2 Department of Radiology, Amsterdam University Medical Centers, Location AMC, Amsterdam, The Netherlands; 3 Department of Surgery, Onze Lieve Vrouwe Gasthuis, Amsterdam, The Netherlands

**Keywords:** trauma, calcaneus, fracture, 3D, 2D, fluoroscopy, imaging, outcome studies

## Abstract

**Background:**

Three-dimensional (3D) fluoroscopy is thought to be advantageous in the open reduction and internal fixation (ORIF) of calcaneal fractures. The goal of this multicenter randomized controlled trial was to investigate the clinical effect of additional intraoperative 3D fluoroscopy on postoperative quality of reduction and fixation and patient-reported outcome as compared to conventional 2-dimensional (2D) fluoroscopy in patients with intra-articular fractures of the calcaneus.

**Methods:**

Patients were randomized to 3D or conventional 2D fluoroscopy during operative treatment of calcaneal fractures. Primary outcome was the difference in quality of fracture reduction and implant position on postoperative computed tomography (CT). Secondary endpoints included intraoperative corrections (prior to wound closure), complications, and revision surgery (after wound closure). Function and patient-reported outcome were evaluated after surgery and included range of motion, Foot and Ankle Outcome Score (FAOS), American Orthopaedic Foot & Ankle Society (AOFAS) score, Short-Form 36 (SF-36) questionnaires, and Kellgren-Lawrence posttraumatic osteoarthritis classification. A total of 102 calcaneal fractures were included in the study in 100 patients. Fifty fractures were randomized to the 3D group and 52 to the 2D group.

**Results:**

There was a statistically significant difference in duration of surgery between the groups (2D 125 min vs 3D 147 min; *P* < .001). After 3D fluoroscopy, a total of 57 intraoperative corrections were performed in 28 patients (56%). The postoperative CT scan revealed an indication for additional revision of reduction or implant position in 69% of the 3D group vs 60% in the 2D fluoroscopy group. At 2 years, there was no difference in number of revision surgery, complications, FAOS, AOFAS score, SF-36 score, or posttraumatic osteoarthritis.

**Conclusion:**

The use of intraoperative 3D fluoroscopy in the treatment of intra-articular calcaneal fractures prolongs the operative procedures without improving the quality of reduction and fixation. There was no benefit of intraoperative 3D fluoroscopy with regard to postoperative complications, quality of life, functional outcome, or posttraumatic osteoarthritis.

**Level of Evidence:** Level I, prospective randomized controlled study.

Displaced intra-articular calcaneal fractures are commonly treated by open reduction and internal fixation (ORIF).^1,6^ The goal of operative treatment is to restore functional anatomy, as intra-articular incongruence leads to poor clinical outcome due to posttraumatic osteoarthritis of the subtalar joint.^15,27,28,34^ Despite the efforts to restore anatomy, up to 20% of operatively treated patients show a persistent step-off in the subtalar joint of >2 mm.^2,7,17^

Intraoperative 2-dimensional (2D) fluoroscopy is used to evaluate the quality of reduction and implant position during ORIF of calcaneal fractures. Due to the complex anatomy of the calcaneus and the subtalar joint, however, conventional fluoroscopy might not always provide sufficient insight.^15,35^ Three-dimensional (3D) fluoroscopy involves a mobile C-arm unit, modified to provide motorized rotational movement combined with a computer workstation. The system provides multiplanar 3D reconstructions of bony structures in addition to conventional 2D fluoroscopic images. The diagnostic accuracy of 3D fluoroscopy appears to be higher than 2D fluoroscopy and similar to computed tomography (CT) for the evaluation of both reduction and implant position.^5,13,22,36^

Three-dimensional fluoroscopy has proven to be a valuable addition to conventional intraoperative fluoroscopy in calcaneal fracture surgery.^16^ Previous studies of 3D fluoroscopy in calcaneal fracture surgery have reported an intraoperative correction rate of up to 47% for indications that were not recognized on conventional 2D fluoroscopy.^14,15,24,35^ The effect of these corrective measures on the radiological and patient-reported outcome has been not been investigated yet.^14,18^ The objective of this study was to investigate the clinical effect of additional intraoperative 3D fluoroscopy on postoperative quality of reduction and fixation and patient-reported outcome as compared to conventional 2D fluoroscopy in patients with intra-articular fractures of the calcaneus.

## Methods

This multicenter randomized clinical trial was conducted in 2 academic level 1 trauma centers and 1 regional teaching hospital between December 2010 and July 2014, with a 2-year follow-up, as described in our published study protocol.^4^ Patients were eligible to participate if they sustained an intra-articular fracture of the calcaneus that required open reduction and internal fixation. Patients were included if they were older than 17 years and signed informed consent was obtained. Patients with bilateral fractures were allowed to participate with both extremities evaluated. Patients were excluded in case of pregnancy, a history of rheumatoid arthritis, or inability to comprehend the trial’s features.

Our sample size calculation was based on the available literature at 2009. The frequency of suboptimal fracture reduction of intra-articular fractures of the wrist, ankle, and calcaneus was 18% to 26%.^9,25,32^ Research in our own hospital, based on postoperative X-rays, showed a frequency of 17% (Weide vd A, Haverlag R, Goslings JC, unpublished data). Based on Kendoff et al,^23^ we anticipated that a suboptimal fracture reduction and/or fixation would be found in 5% of the patients when using the 3D-RX-system. To detect a difference of 12% using a 2-group continuity-corrected χ^2^ test at α = 0.05 and a power of β = 0.80, we had to include 122 patients per randomization group. To account for an approximately 3% dropout by technical or logistic failures of the 3D-RX-system, 250 patients needed to be included for each fracture type.

Reduction and internal fixation were performed through an extended lateral approach (ETA) or sinus tarsi approach (STA), according to the surgeons’ preference. Choice of implants was at the surgeon’s discretion. The study consisted of 2 distinct parts. In the first part, 2D fluoroscopy was available for imaging throughout the operation until the surgeon was satisfied with the reduction and implant position. Prior to wound closure, a 3D fluoroscopy scan was performed in all patients. Whether or not the intraoperative 3D images were to be made available to the surgeon was based on randomization. A dedicated and secured online randomization module performed block randomization stratified for participating center. Patients remained unaware of the availability of the 3D scan to the surgeon throughout the entire trial. In case the results of the 3D fluoroscopy were not made available, the surgeon ended the procedure by wound closure. If the results of 3D fluoroscopy were made available to the surgeon, the surgeon was asked to evaluate the available 3D images according to a scoring protocol for anatomical reduction and implant position, which was published previously.^3,11^ This protocol, based on Delphi consensus, specified 5 categories (23 individual points) to evaluate postoperative reduction of the most important anatomical landmarks of the calcaneus as well as hardware positioning. Corrections were performed (if deemed necessary and feasible) and registered accordingly, after which an additional 3D fluoroscopy scan was performed and evaluated in a similar fashion.

Postoperative CT scans were obtained within 7 days of surgery in all cases. Follow-up outpatient clinic visits were planned for 6 and 12 weeks and 1 and 2 years postoperatively. The postoperative CT scans were anonymized and systematically evaluated by 3 independent blinded observers (an experienced foot and ankle surgeon, a musculoskeletal trauma radiologist, and a PhD candidate with 4 years of research experience in calcaneal fractures). This systematic evaluation by the independent observers was performed at least 6 months after inclusion of patients in the study and did not influence clinical practice. For evaluation of the quality of fracture reduction and fixation and whether a revision was indicated, the previously mentioned imaging 23-question scoring protocol was used.^3,11^ Intra-articular gaps and steps measuring up to 2 mm were deemed acceptable.^11^ A revision was indicated when one of the items was scored as “not acceptable.” An indication for a revision was based only on the radiological evaluation. Technical difficulties, duration of the operation, or other reasons not to perform a revision were not taken into account in the evaluation by the independent observers. Answers of the 3 blinded observers on these 23 items, as well as the indication for a revision in reduction and/or fixation, were combined into a single radiological “profile” of the fracture and implants. In case of inconsistency between observers, majority consensus was sought.

Primary outcome was the need for revision surgery as determined by the observers, based on the postoperative CT scan as described above. Secondary outcomes were the number and type of corrections prior to wound closure after 2D and 3D fluoroscopy, complications, revision operations within 1 year, Foot and Ankle Outcome Score (FAOS), American Orthopaedic Foot & Ankle Society (AOFAS) hindfoot score,^10^ and Short Form 36 (SF-36) questionnaire. Posttraumatic osteoarthritis was classified according to the Kellgren and Lawrence^21^ classification at 2 years postoperatively by 3 independent observers. Total fluoroscopy time is given in seconds, and total radiation dose is given as a dose area product (DAP) in mGy*cm^2^. Previously published power calculations have shown a sample size of 250 patients (125 patients in both arms) for this trial.^4^

The BV Pulsera 3D-RX (Philips Healthcare) mobile C-arm unit prepared for motorized rotational movement for volumetric acquisition and a Philips 3D-RA workstation for visualization of the 3D data set were used in all participating centers. A series of 225 projection images was acquired over a period of 30 seconds during a 200-degree rotation of the C-arm. Both volume rendering and multiplanar reformations (MPRs) in axial, coronal, and sagittal planes were available for evaluation if randomized for allocation in the 3D group.

Statistical analyses were performed in accordance with the intention-to-treat principle using software (SPSS 20.0 for Windows; SPSS, Inc). The primary dichotomous outcome, indication for revision yes/no, and the number of intraoperative corrections based on available 3D fluoroscopy were described as a percentage in both groups. Differences between groups were given as a risk ratio (RR) and risk difference (RD). Scores of functional outcomes were expressed as means and standard deviations (SDs) in case of normal distribution; nonnormally distributed data were expressed as medians with ranges. Continuous parameters were analyzed using the Student *t* test (parametric data) or the Mann-Whitney *U* test (nonparametric data).

Based on a previous study by Agren and colleagues,^1^ an additional subgroup analysis was performed. We selected the patients with the highest 50% AOFAS scores at 2 years postoperatively and performed a logistic regression analysis on age, fracture type (Sanders classification), open fractures, infections, and the availability of 3D fluoroscopy. We repeated this analysis for arthrodesis at 2 years postoperatively.

This study was reported according to the principles of the Consolidated Standards of Reporting Trials (CONSORT) statement guidance. Approval was obtained from the medical ethics committee, and all patients provided written informed consent. The study was registered under Dutch Trial Register NTR 1902.

## Results

Between December 2010 and July 2014, a total of 102 fractures (ie, patients) in 100 patients were included in the study (Suppl. Figure S1). Demographics are displayed in Table 1. Study inclusion ended prior to reaching the expected 250 inclusions due to a lower than predicted accrual rate and subsequent budgetary restraints. No patient withdrew consent. Four patients (5 calcaneal fractures) were lost to follow-up at 12 months postoperatively (2 patients in the 2D group and 2 patients [3 fractures] in the 3D group). In 81 (79.4%) cases, an extended lateral approach (ELA) was used; in 20 (19.6%) cases, the STA was used, and 1 calcaneal fracture (1%) underwent closed reduction and percutaneous fixation.

**Table 1.** Demographics.

Characteristic	2D (50 patients), No. (%)	3D (50 patients), No. (%)	Mean difference (95% CI)	Risk ratio (95% CI)	Risk difference (95% CI)
Including hospital (number of treated fractures)				0.96 (0.70 to 1.32)	−1.31 (–11.87 to 9.25)
I	45 (86.5)	44 (88)		1.02 (0.88 to 1.18)	1.46 (–11.47 to 14.39)
II	5 (9.6)	3 (6.0)		0.62 (0.15 to 2.47)	−3.61 (–13.98 to 6.75)
III	2 (3.8)	3 (6.0)		0.69 (0.12 to 3.98)	−1.76 (–10.12 to 6.58)
Sex, male	39 (75)	42 (84)		1.12 (0.91 to 1.37)	9 (–6.5 to 24.55)
Age, mean (SD)	47.3 (13.4)	45.6 (12.4)	1.7 (–3.4 to 6.8)		
Trauma mechanism					
Fall	12 (23.1)	10 (20.0)		0.87 (0.41 to 1.82)	−3.08 (–19.02 to 12.86)
Fall from height	38 (73.1)	37 (74.0)		1.01 (0.80 to 1.28)	0.92 (–16.2 to 18.04)
Motor vehicle accident	2 (3.8)	1 (2.0)		0.52 (0.05 to 5.56)	−1.85 (–8.36 to 4.66)
Other	0 (0.0)	2 (2.0)		n/a	3.48 (–1.38 to 9.07)
Concomitant injuries	10 (19.2)	17 (34.0)		1.77 (0.90 to 3.48)	14.77 (–2.18 to 31.71)
Fracture ipsilateral lower extremity	3 (5.8)	2 (4.0)		0.69 (0.12 to 3.98)	−1.76 (–10.12 to 6.58)
Fracture contralateral lower extremity	5 (9.6)	6 (12.0)		1.25 (0.41 to 3.83)	2.39 (–9.67 to 14.44)
Left-sided fracture	26 (50.0)	25 (50.0)		1.00 (0.68 to 1.47)	0.00 (–19.41 to 19.41)
Open fracture	1 (2.0)	2 (4.1)		2.08 (0.20 to 22.23)	2.12 (–4.60 to 8.84)
Sanders fracture type				1.08 (0.79 to 1.48)	2.21 (–3.25 to 7.66)
1	1 (1.9)	2 (4.0)		2.08 (0.19 to 22.22)	2.08 (–4.51 to 8.67)
2	18 (34.6)	18 (36.0)		1.04 (0.61 to 1.76)	1.39 (–17.17 to 19.94)
3	24 (46.2)	23 (46.0)		1.00 (0.65 to 1.52)	−0.15 (–19.50 to 19.20)
4	9 (17.3)	7 (6.9)		1.34 (0.54 to 3.32)	4.54 (–9.58 to 18.66)

Abbreviations: 2D, 2-dimensional; 3D, 3-dimensional; n/a, not applicable.

Of the 102 patients, 50 were randomized to intraoperative availability of the 3D fluoroscopy (prior to wound closure); 52 patients were operated on with conventional 2D fluoroscopy alone (3D imaging was obtained but not available to the surgeon). Baseline characteristics were equally distributed among the randomization groups (Table 1). In 3 patients allocated to the 3D group, the 3D system was not available due to a technical error. Subsequently, 47 patients remained for analysis, of whom 28 (56.0%) underwent corrections after 3D images had been reviewed by the surgeon prior to wound closure. Most corrective measures (91.2%) aimed to enhance implant position, of which details are shown in Table 2. Further fracture reduction was performed in 5 (8.8%) patients after availability of 3D fluoroscopy images.

**Table 2.** Operation Characteristics, Intraoperative Imaging, Corrections, and Radiologic Outcome.

Characteristic	2D, No. (%)	3D, No. (%)	*P* value
Days to surgery, median (range)	18.50 (2.0-60.0)	18.12 (4.0-72.0)	.43
Approach	43 (82.7)		.508
Extended lateral approach	9 (17.3)	38 (76.0)	
Sinus tarsi	0 (0.0)	10 (20.0)	
Closed reduction internal fixation		1 (2.0)	
Duration of surgery, median (range), min	125 (69-219)	147 (76-507)	.00
Excluding outlier	125 (69-219)	147 (76-233)	.00
Radiation dose, median (range)			
mGy	3.60 (1.63-9.74)	4.36 (1.44-10.40)	.20
mGy-m^2^	0.06 (0.03-2.25)	0.07 (0.03-0.21)	.04
Time (s)	100 (28-260)	105 (50-274)	.28
Total corrections after 3D		57 (100)	
Reduction			
Step-off	n/a	0 (0.0)	
Gap	n/a	2 (3.5)	
Bone fragment	n/a	2 (5.3)	
Other	n/a	1 (1.8)	
Total		5 (8.8)	
* *Implant position			
Screw too long	n/a	48 (84.2)	
Screw too short	n/a	1 (1.8)	
Screw direction/position	n/a	3 (5.3)	
Plate position	n/a	0 (0.0)	
Total	n/a	52 (91.2)	
3D-based surgeon verdict			
Inadequate reduction	n/a	4 (8.5)	
Inadequate implant position	n/a	3 (6.5)	
Total inadequate ORIF	n/a	7 (15.2)	
Reasons revision not performed			
Inadequate bone quality	n/a	1	
Screw size not in stock	n/a	1	
Reason unspecified	n/a	5	
Total	n/a	7 (15.2)	

Abbreviations: ORIF, open reduction internal fixation; 2D, 2-dimensional; 3D, 3-dimensional; n/a, not applicable.

Radiation dose did not differ in terms of mGy and radiation time. However, the median mGy-m^2^ differed significantly with a median of 0.06 mGy (range, 0.03-2.25) in the 2D group compared to 0.07 mGy (range, 0.03-0.21).

**Figure 1.**
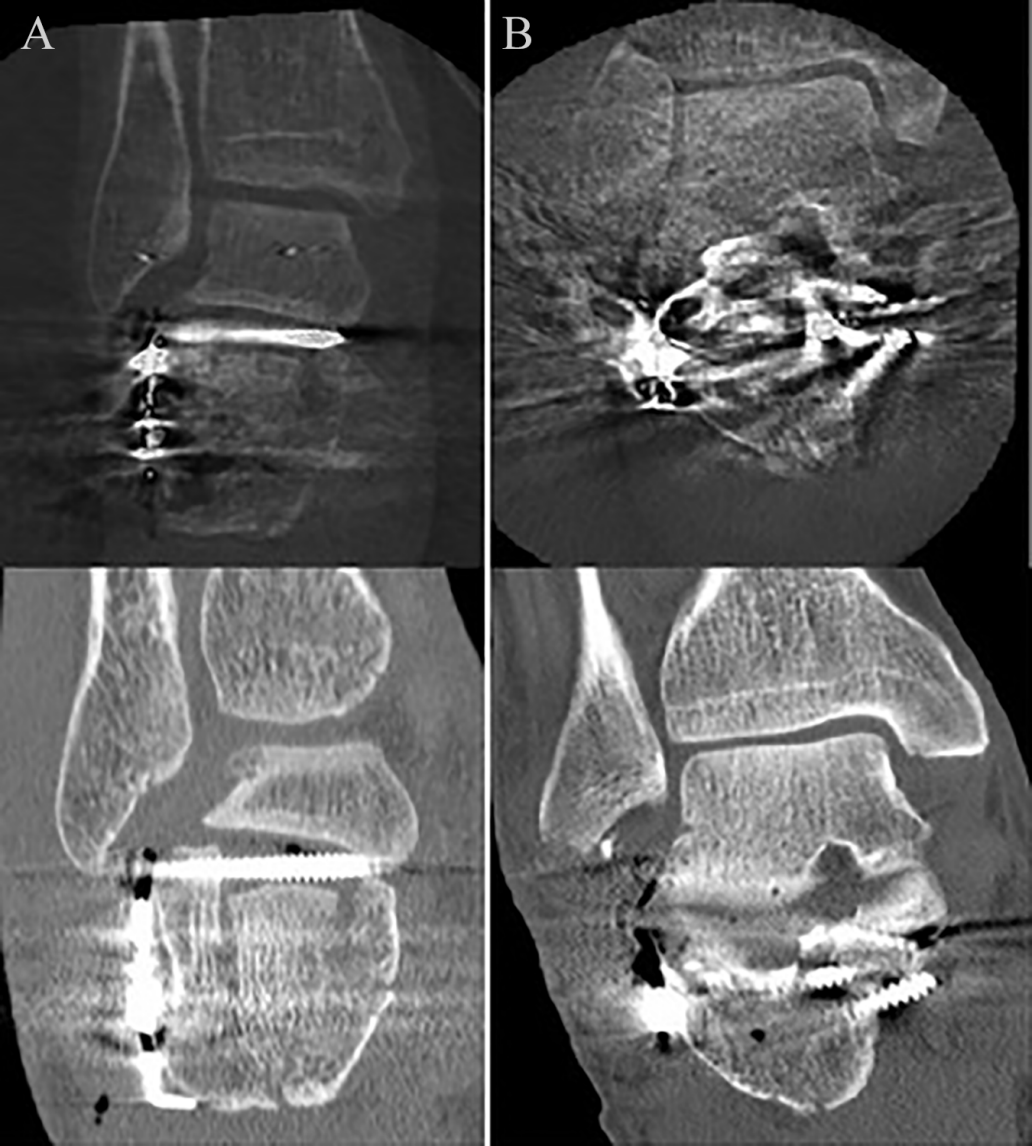
Three-dimensional (3D) fluoroscopy compared to computed tomography (CT) scanning. (A) This patient was randomized in the conventional 2-dimensional (2D) fluoroscopy group. The upper image is the 3D scan. The postoperative CT scan (bottom image) clearly showed an unacceptable reduction of the posterior talocalcaneal joint and an intra-articular screw position; both findings were also recognized on the postoperative evaluation of the 3D fluoroscopy. Patient underwent revision surgery within 24 hours and had suffered from a superficial wound infection. (B) This patient was randomized to the 3D fluoroscopy group. The 3D images (upper image), however, show substantial scattering, impeding proper evaluation of the images. The postoperative CT scan (bottom image) showed a medially protruding screw that missed the sustentaculum.

The postoperative CT scan as evaluated by 3 independent observers revealed an indication for additional revision of reduction or implant position in 69.4% of the 3D group vs 59.6% in the 2D fluoroscopy group. The corresponding risk ratio of 1.16 (95% CI, 0.87-1.56) did not reach statistical significance. Revision of reduction or fixation as suggested by the raters was performed in 3 patients. In 1 patient in the 3D scan group, an intra-articular screw was revised. In the 2D group, 2 revision operations were performed, one because of an insufficient reduction of the posterior talocalcaneal joint, and in another patient, an intra-articular screw was revised. In 7 patients, indications for corrective measures were identified postoperatively and also identified intraoperatively but not performed for various reasons (Table 2). Examples of intraoperative 3D and corresponding postoperative CT images are shown in Figure 1.

There was a statistically significant difference in duration of surgery between the groups, with a median of 147 minutes (3D group) vs 125 minutes (2D group) (*P* < .001). Exclusion of the 1 outlier in the 3D group with 507 minutes due to operative treatment of concomitant injuries did not change this result.

There were no significant differences between groups in terms of type of approach, revision surgery, complications, wound infections, posttraumatic osteoarthritis, short-term rate of arthrodesis, and patient-reported outcome measures including AOFAS score, FAOS, and SF-36 score (Table 3). Patient-reported outcomes are shown in Supplemental Table S1.

**Table 3.** Complications, Posttraumatic Arthritis, and Arthrodesis.

Characteristic	2D, No. (%)	3D, No. (%)	Risk ratio (95% CI)	Risk difference (95% CI)
Revision required CT-based outcome independent observer	31 (59.6)	34 (69.4)	1.16 (0.87 to 1.56)	9.77 (–8.78 to 28.33)
ELA	27 (51.9)	25 (48.1)	1.08 (0.78 to 1.48)	4.78 (–16.11 to 25.66)
STA	4 (44.4)	9 (81.8)	1.84 (0.84 to 4.02)	37.7 (–2.29 to 77.04)
Revision surgery (1 year)	20 (38.5)	17 (34.0)	0.88 (0.53 to 1.48)	−4.46 (–23.1 to 14.17)
ELA	17 (39.5)	13 (34.2)	0.87 (0.49 to 1.54)	−5.32 (–26.33 to 15.68)
STA	3 (33.3)	4 (36.4)	1.09 (0.33 to 3.66)	3.03 (–38.88 to 44.94)
Deep infection with debridement	6 (11.5)	4 (8.0)		
Deep infection with hardware removal	3 (5.8)	4 (8.0)		
Reduction and/or fixation	1 (1.9)	1 (2.0)		
Implant removal (planned)	0 (0.0)	1 (2.0)		
Implant removal (complaints)	9 (17.3)	7 (14.0)		
First infection with debridement, second surgery with implant removal	1 (1.9)	2 (4.0)		
Infectious complications	14 (27.5)	11 (22.9)	0.82 (0.41 to 1.63)	−4.92 (–21.57 to 11.72)
ELA	13 (31.0)	10 (27.0)	0.87 (0.44 to 1.75)	−3.92 (–23.93 to 16.08)
STA	1 (11.1)	0 (0.0)	n/a	−11.11 (–31.64 to 9.42)
CRIF	0 (0.0)	1 (100)	n/a	n/a
Superficial without antibiotics	0 (0.0)	2 (4.2)	n/a	4.00 (–1.43 to 9.43)
Superficial with antibiotics	4 (7.8)	1 (2.1)	0.26 (0.03 to 2.25)	−5.69 (–13.91 to 2.52)
Deep with debridement	7 (13.7)	4 (8.3)	0.59 (0.19 to 1.91)	−5.46 (–17.40 to 6.48)
Deep with hardware removal	3 (5.9)	4 (8.3)	1.39 (0.33 to 5.89)	2.23 (–7.60 to 12.06)
Posttraumatic arthritis^a^ (2 years)				
0	2 (3.8)	1 (2.0)	0.53 (0.05 to 5.57)	−2.49 (–11.39 to 6.42)
1	7 (13.5)	5 (19,2)	1.51 (0.64 to 3.53)	9.36 (–9.77 to 28.49)
2	12 (23.1)	8 (16.0)	0.70 (0.33 to 1.52)	−9.36 (–29.43 to 0.71)
3	12 (23.1)	10 (20.0)	0.88 (0.43 to 1.78)	−3.80 (–24.6 to 16.99)
4	5 (9.6)	7 (14.0)	1.48 (0.52 to 4.24)	6.29 (–10.52 to 23.1)
Missing	14 (26.9)	14 (28.0)		
Arthrodesis (2 years)	1 (2.0)	5 (10.2)	5 (0.61 to 41.2)	8.16 (–1.19 to 17.5)
ELA	0 (0.0)	5 (14.3)	n/a	14.29 (2.69 to 25.88)
STA	1 (11.1)	0 (0.0)	n/a	−11.11 (–31.64 to 9.42)

Abbreviations: CRIF, closed reduction and internal fixation; CT, computed tomography; ELA, extended lateral approach; n/a, not applicable; STA, sinus tarsi approach; 2D, 2-dimensional; 3D, 3-dimensional.

a Classification according to Kellgren and Lawrence.^21^

Although infectious complications occurred more when ELA (29.1%) was used compared to STA (5.3%) (RR, 0.18 [0.03-1.26]; RD, –23.85 [–38.03 to −9.67]), additional subgroup regression analysis showed no association between 50% of patients with the highest AOFAS score at 2 years postoperatively and age, fracture type, open fractures, type of approach, infections, availability of 3D fluoroscopy, or duration of operation. Furthermore, we found no association for these factors with arthrodesis at 2 years postoperatively.

## Discussion

Despite 57 individual intraoperative corrections in 28 patients (56% of the 3D group), the current study did not find a beneficial effect of intraoperative 3D fluoroscopy in terms of radiological, patient-reported, or functional (eg, range of motion) outcome as compared to conventional 2D fluoroscopy. Follow-up CT scan revealed indications for revision regardless of prior availability of 3D fluoroscopy images during surgery and performed corrections. Moreover, the duration of the surgical procedure was significantly longer in the 3D group.

To our knowledge, this is the first randomized controlled trial reporting the functional results of patients in which additional 3D fluoroscopy was compared to conventional fluoroscopy in the treatment of calcaneal fractures. In 2015, Gwak et al^18^ published a retrospective cohort study of 60 calcaneal fractures, half of which were treated with additional 3D fluoroscopy. In accordance with our results, they found no statistically significant differences between groups in terms of Böhler angle, Gissane angle, AOFAS score, or visual analog scale pain score after 2 years postoperatively.

Most other available studies reporting on 3D fluoroscopy lack a control group or put emphasis on the number of intraoperative 3D-related corrections rather than reporting functional or radiological outcome.^12,15,24,35^ In 2015, Eckhardt et al^12^ published a series of 62 calcaneal fractures operated on using intraoperative 3D imaging. They used an O-arm with high-quality imaging, leading to 40% corrections and good radiological results on the final intraoperative 3D scan. No postoperative CT scan was made as a gold standard; they did not have a control group with conventional fluoroscopy or report functional outcome. In 2014, Franke et al^15^ published a large retrospective cohort of operatively treated calcaneal fractures using 3D fluoroscopy and showed an intraoperative correction rate of 40.3%. Of the evaluated group, 45% still had residual step-off of ≥2 mm on the postoperative evaluation of the 3D scan. No control group was mentioned in terms of 2D fluoroscopy.

Our results show considerable percentages of indications for revision based on the postoperative CT scan. Multiple factors potentially contribute to these high revision rates. First, we evaluated 23 items of reduction and fixation per patient. These items included Böhler and Gissane angles, as well as steps, gaps, and bone fragments of the posterior talocalcaneal, calcaneocuboid, and anterior talocalcaneal joints. In addition, the position of fixation material was scored in the previously mentioned joints and the sustentaculum tali and medial wall. When scoring to such an extent, instead of solely focusing on, for example, the joint surface, one is more likely to find indications for improvement. Moreover, images were often difficult to interpret due to the amount of scattering caused by the implants regardless of software used. Third and most important, the evaluation of our CT images was done outside of the operation room. Consequently, observers were not hampered by the reality of operative challenges, additional risks of further surgical procedures, and time constraints, lowering the threshold for finding indications for implant and reduction improvement.

The indicated revisions were identified by the operating surgeons in only 10 patients, and only 3 of them were actually revised. The postoperative CT scans were evaluated by the operating surgeon but not scored by them according to the 23-item scoring list. Therefore, we do not know whether the other indicated revisions were also identified by the operating surgeons. Reasons for the much lower actual revision rate could have been the operating surgeons did not agree with the rater’s indications for revision. Other reasons could be lack of bone stock, technical challenges, or risk of wound infection by a second operation.

Despite the high percentage of indicated revisions, functional results of our cohort are comparable to the literature. In 2009, Kienast et al^24^ used 3D fluoroscopy in a series of 136 operatively treated calcaneal fractures. At an average follow-up of 8.6 months, the average AOFAS score was between 81 and 84. The previously mentioned study by Gwak et al^18^ reported average AOFAS scores between 78.3 and 82.3 after a 2-year follow-up. The minimal clinically important difference (MCID) of the AOFAS score following calcaneal fracture surgery is not known, but the AOFAS difference between 2D and 3D groups is well below the known MCID for hallux valgus surgery (7.9 points).^8^ SF-36 scores are comparable to other large clinical trials.^2,17^

In this study, there was an indication for a revision in, respectively, 69.4% of the 3D group vs 59.6% in the 2D group. This indication for revision rate almost triples the 20% described in the literature.^2,7,17^ The reason for this difference is that we performed an extensive evaluation of the postoperative CT scan, while most of the mentioned studies based their indications for revision on postoperative X-rays. Not all articular incongruencies and misplaced fixation material found in CT scans are detected on X-rays.

In addition, the strict evaluation of the postoperative CT scan showed no relation with the postoperative clinical outcome or incidence of 2-year posttraumatic arthritis. This could be due to our limited number of patients and the variety of incidence of incongruencies in the reduction and fixation of the calcaneal fractures. Another reason could be that the indications for revisions based on the postoperative CT scan were too strict.

In our study, 24.5% of patients had a postoperative wound infection, which is quite high but also encountered in other studies.^2,12,17^ The large number of extended lateral approaches was responsible for the more than 20% of wound complications, comparable to the literature.^26,31^ Although there is a shift to the use of the sinus tarsi approach, the extended lateral approach has not been abandoned completely.^20^ Even though there is a difference in infectious complications between ELA and STA, Schepers et al^31^ showed there are no differences in radiological outcome between the 2 approaches. In addition, the type of approach was not related to patient-relevant outcome or posttraumatic arthritis in our study.

A strength of this study is that we were able to evaluate clinical effectiveness of this technique by comparison of an intervention (3D) and a control group (2D). Not only were we able to obtain validated functional outcome parameters, but we also systematically evaluated reduction and hardware position on CT using a detailed protocol. Instead of exact measurements that are mostly performed in research settings, we have used subjective evaluations (eg, good, moderate, or poor). This approach mimics intraoperative evaluation. During surgery, no measurements (eg, Böhler angle measurement) can be performed—the surgeon can only eyeball the quality of reduction and fixation, based on his or her experience with the acceptable measurements. Moreover, subjective (categorical) and objective (numerical values) evaluations have previously proven to have a good correlation.^19^

Limitations of this study include that as the project progressed, surgeons became more accustomed to the use of 3D fluoroscopy techniques. Inspired by the benefits of multiple-angle views, surgeons sporadically used continuous fluoroscopy while turning the foot manually. This maneuver potentially provided additional information, leading to more radiation exposure, and reduced the additional value of 3D fluoroscopy. Even though study inclusion was ended prior to reaching the expected 250 inclusions for the primary radiological outcome, no trend toward clinically relevant differences was seen. Therefore, we do not believe results would have been different if we had included more patients. Our power calculation was based on a suboptimal reduction and fixation of only 17% based on postoperative X-rays. As we can identify more suboptimal aspects in reduction and fixation based on a CT scan, first a definition of CT-based indications for revisions should have been developed to perform a proper power analysis.

This study was designed with analysis of the diagnostic accuracy of 3D fluoroscopy in mind. For this purpose, both randomization groups were subject to 3D fluoroscopy. As the radiation dose of a single 3D scan is different for each individual patient, we were not able to correct for the received 3D scan in the 2D group. Hence, the additional radiation dose in the 3D group as mentioned in Table 2 is a consequence of fluoroscopy (2D and/or 3D) used *after* the initial 3D scan. The maximum equivalent dosage of a 3D-RX scan of the extremities is 17 µSv. Although more 3D scans were performed in the 3D group, no clinically relevant difference could be seen between groups in terms of radiation exposure. Unfortunately, we cannot extract the radiation dose used for 2D fluoroscopy alone and the fluoroscopy used for the 3D run. However, this suggests that the additional 2D fluoroscopy dosage used for 2D images is comparable to the radiation dose of a 3D scan. Additional radiation exposure for the patient and personnel can be classified as “minor risk” according to the International Commission on Radiological Protection (ICRP) (report ICRP62).

The radiation exposure is expressed as DAP in mGy*cm^2^. We chose to refrain from estimating effective dose (mSv) because of its uncertain reliability.^30,33^ Rausch et al^29^ reported a mean DAP of 392 ± 145 mGy/cm^2^ for 3D fluoroscopy in a series of operatively treated wrist fractures. Our 3D group received a median of 726 mGy/cm^2^. The bigger mass of the lower extremity is accountable for a large part of this difference in radiation dose.

With high percentages of intraoperative corrections, mainly implant related, it is likely that 3D fluoroscopy has some form of advantage. Future studies should elucidate and specify these advantages, potentially by narrowing down the indications for use of this technique. Calcaneal fractures that are particularly at risk for medial or intra-articular screw protrusion might benefit more from 3D fluoroscopy than fractures that need less complex fixation.

## Conclusion

The use of intraoperative 3D fluoroscopy prolonged the procedure without improving the quality of reduction and fixation in the management of calcaneal fractures. We found no benefit of intraoperative 3D vs 2D fluoroscopy with regard to postoperative complications, quality of life, functional outcome, or posttraumatic osteoarthritis at 2-year follow-up.

## Supplemental Material

FAI926111_disclosures – Supplemental material for Intraoperative Effect of 2D vs 3D Fluoroscopy on Quality of Reduction and Patient-Related Outcome in Calcaneal Fracture Surgery Click here for additional data file. Supplemental material, FAI926111_disclosures for Intraoperative Effect of 2D vs 3D Fluoroscopy on Quality of Reduction and Patient-Related Outcome in Calcaneal Fracture Surgery by Jens A. Halm, M. Suzan H. Beerekamp, Robert Jan de Muinck-Keijzer, Ludo F. M. Beenen, Mario Maas, J. Carel Goslings and Tim Schepers in Foot & Ankle International

Supplemental_Figure_S1_Flow_chart – Supplemental material for Intraoperative Effect of 2D vs 3D Fluoroscopy on Quality of Reduction and Patient-Related Outcome in Calcaneal Fracture Surgery Click here for additional data file. Supplemental material, Supplemental_Figure_S1_Flow_chart for Intraoperative Effect of 2D vs 3D Fluoroscopy on Quality of Reduction and Patient-Related Outcome in Calcaneal Fracture Surgery by Jens A. Halm, M. Suzan H. Beerekamp, Robert Jan de Muinck-Keijzer, Ludo F. M. Beenen, Mario Maas, J. Carel Goslings and Tim Schepers in Foot & Ankle International

Supplementary – Supplemental material for Intraoperative Effect of 2D vs 3D Fluoroscopy on Quality of Reduction and Patient-Related Outcome in Calcaneal Fracture Surgery Click here for additional data file. Supplemental material, Supplementary for Intraoperative Effect of 2D vs 3D Fluoroscopy on Quality of Reduction and Patient-Related Outcome in Calcaneal Fracture Surgery by Jens A. Halm, M. Suzan H. Beerekamp, Robert Jan de Muinck-Keijzer, Ludo F. M. Beenen, Mario Maas, J. Carel Goslings and Tim Schepers in Foot & Ankle International

## References

[1] Agren P-H Mukka S Tullberg T Wretenberg P Sayed-Noor AS. Factors affecting long-term treatment results of displaced intra-articular calcaneal fractures: a post-hoc analysis of a prospective, randomized, controlled multicenter trial. J Orthop Trauma. 2014;28(10):564-568. 24824095 10.1097/BOT.0000000000000149

[2] Agren P-H Wretenberg P Sayed-Noor AS. Operative versus nonoperative treatment of displaced intra-articular calcaneal fractures: a prospective, randomized, controlled multicenter trial. J Bone Joint Surg Am. 2013;95(15):1351-1357. 23925738 10.2106/JBJS.L.00759

[3] Beerekamp MSH Luitse JSK Ubbink DT Maas M Schep NWL Goslings JC Evaluation of reduction and fixation of calcaneal fractures: a Delphi consensus. Arch Orthop Trauma Surg. 2013;133(10):1377-1384. 23892557 10.1007/s00402-013-1823-5

[4] Beerekamp MSH Ubbink DT Maas M, et al Fracture surgery of the extremities with the intra-operative use of 3D-RX: a randomized multicenter trial (EF3X-trial). BMC Musculoskelet Disord. 2011;12(1):151. 21733185 10.1186/1471-2474-12-151 PMC3152540

[5] Beerekamp MSHS Sulkers GSI Ubbink DT Maas M Schep NWL Goslings JC Accuracy and consequences of 3D-fluoroscopy in upper and lower extremity fracture treatment: a systematic review. Eur J Radiol. 2012;81(12):4019-4028. 22975150 10.1016/j.ejrad.2012.06.021

[6] Bruce J Sutherland A. Surgical versus conservative interventions for displaced intra- articular calcaneal fractures. Cochrane Database Syst Rev. 2013;(1):CD008628. 10.1002/14651858.CD008628.pub2 23440830

[7] Buckley R Tough S McCormack R, et al Operative compared with nonoperative treatment of displaced intra-articular calcaneal fractures: a prospective, randomized, controlled multicenter trial. J Bone Joint Surg Am. 2002;84(10):1733-1744. 12377902 10.2106/00004623-200210000-00001

[8] Chan HY Chen JY Zainul-Abidin S Ying H Koo K Rikhraj IS. Minimal clinically important differences for American Orthopaedic Foot & Ankle Society score in hallux valgus surgery. Foot Ankle Int. 2017;38(5):551-557. 28193121 10.1177/1071100716688724

[9] Chen S-H Wu P-H Lee Y-S. Long-term results of pilon fractures. Arch Orthop Trauma Surg. 2007;127(1):55-60. 17004076 10.1007/s00402-006-0225-3

[10] Coster MC Rosengren BE Bremander A Brudin L Karlsson MK. Comparison of the Self-reported Foot and Ankle Score (SEFAS) and the American Orthopaedic Foot & Ankle Society Score (AOFAS). Foot Ankle Int. 2014;35(10):1031-1036. 25015390 10.1177/1071100714543647

[11] de Muinck Keizer RJO Beerekamp MSH Ubbink DT Beenen LFM Schepers T Goslings JC. Systematic CT evaluation of reduction and hardware positioning of surgically treated calcaneal fractures: a reliability analysis. Arch Orthop Trauma Surg. 2017;137(9):1261-1267. 28748292 10.1007/s00402-017-2744-5 PMC5565655

[12] Eckardt H Lind M. Effect of intraoperative three-dimensional imaging during the reduction and fixation of displaced calcaneal fractures on articular congruence and implant fixation. Foot Ankle Int. 2015;36(7):764-773. 25761853 10.1177/1071100715576518

[13] Euler E Wirth S Linsenmaier U Mutschler W Pfeifer KJ Hebecker A. Comparative study of the quality of C-arm based 3D imaging of the talus. Unfallchirurg. 2001;104:839-846. 11572126 10.1007/s001130170055

[14] Franke J von Recum J Wendl K Grützner P. Intraoperative 3-dimensional imaging—beneficial or necessary [in German]? Unfallchirurg. 2013;116(2):185-190. 23404358 10.1007/s00113-013-2359-4

[15] Franke J Wendl K Suda A Giese T Grützner P von Recum J. Intraoperative three-dimensional imaging in the treatment of calcaneal fractures. J Bone Jt Surg. 2014;96(72):1-7. 10.2106/JBJS.L.01220 24806018

[16] Geerling J Kendoff D Citak M, et al Intraoperative 3D imaging in calcaneal fracture care-clinical implications and decision making. J Trauma. 2009;66(3):768-773. 19276751 10.1097/TA.0b013e31816275c7

[17] Griffin D Parsons N Shaw E, et al Operative versus non-operative treatment for closed, displaced, intra-articular fractures of the calcaneus: randomised controlled trial. BMJ. 2014;349:1-13. 10.1136/bmj.g4483 PMC4109620 25059747

[18] Gwak H-C Kim J-G Kim J-H Roh S-M. Intraoperative three-dimensional imaging in calcaneal fracture treatment. Clin Orthop Surg. 2015;7(4):483-489. 26640632 10.4055/cios.2015.7.4.483 PMC4667117

[19] Heiney JP Redfern RE Wanjiku S. Subjective and novel objective radiographic evaluation of inflatable bone tamp treatment of articular calcaneus, tibial plateau, tibial pilon and distal radius fractures. Injury. 2013;44(8):1127-1134. 23601366 10.1016/j.injury.2013.03.020

[20] Jansen SCP Bransen J van Montfort G Besselaar AT van der Veen AH Should the extended lateral approach remain part of standard treatment in displaced intra-articular calcaneal fractures? J Foot Ankle Surg. 2018;57(6):1120-1124. 30205938 10.1053/j.jfas.2018.05.015

[21] Kellgren JH Lawrence JS. Radiological assessment of osteo-arthrosis. Ann Rheum Dis. 1957;16(3):494-503. 13498604 10.1136/ard.16.4.494 PMC1006995

[22] Kendoff D Citak M Gardner M, et al Three-dimensional fluoroscopy for evaluation of articular reduction and screw placement in calcaneal fractures. Foot Ankle Int. 2007;28(11):1165-1171. 18021585 10.3113/FAI.2007.1165

[23] Kendoff D Citak M Gardner MJ Stübig T Krettek C Hüfner T. Intraoperative 3D imaging: value and consequences in 248 cases. J Trauma. 2009;66(1):232-238. 19131832 10.1097/TA.0b013e31815ede5d

[24] Kienast B Gille J Queitsch C, et al Early weight bearing of calcaneal fractures treated by intraoperative 3D-fluoroscopy and locked-screw plate fixation. Open Orthop J. 2009;3:69-74. 19750017 10.2174/1874325000903010069 PMC2738828

[25] Langenhuijsen JF Heetveld MJ Ultee JM Steller EP Butzelaar RMJM Results of ankle fractures with involvement of the posterior tibial margin. J Trauma. 2002;53(1):55-60. 12131390 10.1097/00005373-200207000-00012

[26] Nosewicz TL Dingemans SA Backes M Luitse JSK Goslings JC Schepers T. A systematic review and meta-analysis of the sinus tarsi and extended lateral approach in the operative treatment of displaced intra-articular calcaneal fractures. Foot Ankle Surg. 2019;25(5):580-588. 30321924 10.1016/j.fas.2018.08.006

[27] Paley D Hall H. Intra-articular fractures of the calcaneus: a critical analysis of results and prognostic factors. J Bone Joint Surg Am. 1993;75(3):342-354. 8444912 10.2106/00004623-199303000-00005

[28] Pozo JL Kirwan EO Jackson AM. The long-term results of conservative management of severely displaced fractures of the calcaneus. J Bone Joint Surg Br. 1984;66(3):386-390. 6725350 10.1302/0301-620X.66B3.6725350

[29] Rausch S Marintschev I Graul I, et al Tangential view and intraoperative three-dimensional fluoroscopy for the detection of screw-misplacements in volar plating of distal radius fractures. Arch Trauma Res. 2015;4(2):e24622. 26101762 10.5812/atr.4(2)2015.24622 PMC4475339

[30] Saltybaeva N Jafari ME Hupfer M Kalender WA. Effective dose estimates for CT scans of lower extremities. Radiology. 2014;273(1):153-159. 24937693 10.1148/radiol.14132903

[31] Schepers T Backes M Dingemans SA De Jong VM Luitse JSK Similar anatomical reduction and lower complication rates with the sinus tarsi approach compared with the extended lateral approach in displaced intra-articular calcaneal fractures. J Orthop Trauma. 2017;31(6):293-298. 28538451 10.1097/BOT.0000000000000819

[32] Stulik J Stehlik J Rysavy M Wozniak A. Minimally-invasive treatment of intra-articular fractures of the calcaneum. J Bone Joint Surg Br. 2006;88(12):1634-1641. 17159178 10.1302/0301-620X.88B12.17379

[33] Svalkvist A Hansson J Bath M. Estimating effective dose from 3D imaging with interventional fluoroscopy systems using limited exposure data. Acta Radiol. 2016;57(3):356-361. 25852194 10.1177/0284185115579079

[34] Thordarson D Krieger L Operative vs. nonoperative treatment of intra-articular fractures of the calcaneus: a prospective randomized trial. Foot Ankle Int. 1996;17(1):2-9. 8821279 10.1177/107110079601700102

[35] von Recum J Wendl K Vock B Grützner P Franke J Intraoperative 3D C-arm imaging: state of the art [in German]. Unfallchirurg. 2012;115(3):196-201. 22367513 10.1007/s00113-011-2119-2

[36] Weil YA Liebergall M Mosheiff R Singer SB Joskowicz L Khoury A. Assessment of two 3-D fluoroscopic systems for articular fracture reduction: a cadaver study. Int J Comput Assist Radiol Surg. 2011;6(5):685-692. 21298490 10.1007/s11548-011-0548-6

