# Supplementary material for: Intraoperative Effect of 2D vs 3D Fluoroscopy on Quality of Reduction and Patient-Related Outcome in Calcaneal Fracture Surgery
Source: Foot Ankle Int. 2020 Jun 9;41(8):954–63. doi: 10.1177/1071100720926111 (PMC7406967; doi:10.1177/1071100720926111)
Supplement: Supplemental_Figure_S1_Flow_chart – Supplemental material for Intraoperative Effect of 2D vs 3D Fluoroscopy on Quality of Reduction and Patient-Related Outcome in Calcaneal Fracture Surgery [file Supplemental_Figure_S1_Flow_chart.docx]

**Subjects excluded (n=45**)

- No informed consent (n=7)
- Unable to comprehend (n=11)
- Missed (n=9)
- Primary arthrodesis (n=13)
- Logistical problems (n=3)
- Rheumatoid arthritis (n=1)
- Protocol violation (n=1)

**Subjects screened (n=147)** December 2010-June 2014

**Primary outcome**
CT-scan <1 wk postoperative (n=49)

♦ No postoperative CT-scan available (n=1)

**Clinical follow-up**
12 wks (n=48)

♦ 1 patient with a bilateral fracture did not keep appointment

1 yr (n=47)

♦ 2 patients did not keep appointment t, 1 with bilateral fracture

2 yr (n=39)
♦ 10 patients (1 with bilateral fracture) did not keep appointment

## Analysis

## Follow up

## Allocation

**AOFAS**
12 wks (n=25)

♦ 27 not filled in sufficiently to calculate outcome

1 year (n= 28)

♦ 24 not filled in sufficiently to calculate outcome

2 year (n=26)

♦ 26 not filled in sufficiently to calculate outcome

**FAOS**

12 wks (n=35)

♦ 17 not filled in sufficiently to calculate outcome

1 yr (n=33)

♦ 19 not filled in sufficiently to calculate outcome

2 yr (n=31)

♦ 21 not filled in sufficiently to calculate outcome

**SF-36**

12 wks (n=33)

♦ 19 not filled in sufficiently to calculate outcome

1 yr (n=33)

♦ 19 not filled in sufficiently to calculate outcome

2 yr (n=24)
♦ 28 not filled in sufficiently to calculate outcome

**Arthrosis according to Kellgren & Lawrence
after 2 years (n=37)**
♦ 10 patients did not keep appointment

♦ 5 patients did not receive an X-ray at the 2-year appointment

**AOFAS**
12 wks (n=21)

♦ 29 not filled in sufficiently to calculate outcome

1 yr (n= 27)

♦ 23 not filled in sufficiently to calculate outcome

2 yr (n=26)

♦ 24 not filled in sufficiently to calculate outcome

**FAOS**

12 wks (n=33)

♦ 17 not filled in sufficiently to calculate outcome

1 yr (n=33)

♦ 17 not filled in sufficiently to calculate outcome

2 yr (n=29)

♦ 21 not filled in sufficiently to calculate outcome

**SF-36**

12 wks (n=27)

♦ 23 not filled in sufficiently to calculate outcome

1 yr (n=35)

♦ 15 not filled in sufficiently to calculate outcome

2 yr (n=27)
♦ 23 not filled in sufficiently to calculate outcome

**Arthrosis according to Kellgren & Lawrence
after 2 years (n=35)**
♦ 10 patients (11 fractures) did not keep appointment

♦ 4 patients did not receive an X-ray at the 2-year appointment

**Primary outcome**CT-scan <1 wk postoperative (n=52)

**Clinical follow-up**
12 wks (n=52)

1 yr (n=50)

♦ 2 patients did not keep appointment

2 yr (n=42)
♦ 10 patients did not keep appointment

**Fractures allocated to intervention 3D (n=50)**

♦ Received allocated intervention (n=47)

♦ Did not receive allocated intervention

(technical malfunction) (n=3)

**Fractures allocated to control 2D (n=52)**

♦ Received allocated intervention (n=52)

♦ Did not receive allocated intervention (n=0)

**Calcaneal fractures randomized (n=102)**
 in 100 patients

## Enrollment
