# Supplementary material for: Intraoperative Effect of 2D vs 3D Fluoroscopy on Quality of Reduction and Patient-Related Outcome in Calcaneal Fracture Surgery
Source: Foot Ankle Int. 2020 Jun 9;41(8):954–63. doi: 10.1177/1071100720926111 (PMC7406967; doi:10.1177/1071100720926111)
Supplement: Supplementary – Supplemental material for Intraoperative Effect of 2D vs 3D Fluoroscopy on Quality of Reduction and Patient-Related Outcome in Calcaneal Fracture Surgery [file Supplementary.pdf]

Supplementary
Table 4 ROM, patient reported outcomes

	Characteristic		2D n (%)	3D n (%)	P-value
**Dorsi/plantar flexion (°)**					
		1 yr	50.0 (30-80)	55.0 (20-80)	0.44
		2 yr	50.0 (30-90)	55.0 (20-80)	0.43
**In/Eversion (°)**					
		1 yr	15.0 (0-50)	20.0 (0-60)	0.80
		2 yr	7.5 (0-60)	10.0 (0-40)	0.48
**AOFAS, median (range)**					
		1yr	80.5 (54-97)	78.0 (38-97)	0.19
		2 yr	82.0 (46-100)	80.0 (26-100)	0.51
**FAOS, median (range)**	Symptoms				
		1yr	57.1 (18-82)	55.4 (29-82)	0.75
		2 yr	57.1 (29-86)	53.6 (29-79)	0.51
	Pain				
		1yr	65.3 (3-100)	69.4 (39-100)	0.37
		2 yr	75.0 (6-100)	75.0 (28-100)	0.82
	ADL				
		1yr	82.4 (19-100)	79.4 (32-100)	0.94
		2 yr	92.7 (15-100)	86.8 (32-100)	0.50
	Sport/Recreation				
		1yr	40.0 (0-100)	45.0 (0-100)	0.90
		2 yr	65.0 (0-100)	70.0 (0-100)	0.50
	Quality of Life				
		1yr	56.3 (6-100)	43.8 (0-94)	0.34
		2 yr	56.3 (0-94)	86.8 (32-100)	0.88
**SF-36, median (range)**	PCS				
		1yr	43.1 (23-59)	41.9 (27-59)	0.96
		2 yr	48.6 (29-61)	45.2 (27-61)	0.43
	MCS				
		1yr	56.5 (28-65)	41.9 (27-59)	0.17
		2 yr	50.9 (20-60)	52.6 (26-61)	0.71

CI: confidence interval; PCS: physical component scale; MCS: mental component scale;
